# 

**Published:** 1897-04

**Authors:** 


### CONTENTS.

ORIGINAL COMMUNICATIONS. PAGE

A Contribution to the Study of the Development of Dental Enamel. By R. R. Andrews, A.M., D.D.S., F.R.M.S. . . . 205

The Degenerate Jaws and Teeth. (Concluded.) By Eugene S. Talbot, M.D., D.D.S. . . . 225

Union of the American and Southern Dental Associations. By Thomas Fillebrown, M.D., D.M.D., Boston, Mass. . . . 236

The Restoration of Badly Broken Teeth without Crowning. By S. E. Davenport, D.D.S., M.D.S., New York . . . 240

Annual Address. By George A. Maxfield, D.D.S., Holyoke, Mass. . . . 247

ABSTRACTS AND TRANSLATIONS.

Investigations of the Nerves of the Pulp by the Methylene-Blue Method. By Michael Morgenstern, Germany. . . . 255

REPORTS OF SOCIETY MEETINGS.

The New York Institute of Stomatology . . . 263

EDITORIAL.

Reorganization . . . 278

Is it Just? . . . 280

BIBLIOGRAPHY . . . 281

CURRENT NEWS.

Dental Society of the State of New York . . . 282

Harvard Odontological Society . . . 283

Recent Patents . . . 283

SELECTIONS.

Peroxide of Hydrogen in Wound Treatment . . . 284

